# A Non-VH1-69 Heterosubtypic Neutralizing Human Monoclonal Antibody Protects Mice against H1N1 and H5N1 Viruses

**DOI:** 10.1371/journal.pone.0034415

**Published:** 2012-04-04

**Authors:** Donata De Marco, Nicola Clementi, Nicasio Mancini, Laura Solforosi, Guisella J. Moreno, Xiangjie Sun, Terrence M. Tumpey, Larisa V. Gubareva, Vasiliy Mishin, Massimo Clementi, Roberto Burioni

**Affiliations:** 1 Laboratory of Microbiology and Virology, Vita-Salute San Raffaele University, Milan, Italy; 2 Influenza Division, National Center for Immunization and Respiratory Diseases, Centers for Disease Control and Prevention, Atlanta, Georgia, United States of America; National University of Singapore, Singapore

## Abstract

Influenza viruses are among the most important human pathogens and are responsible for annual epidemics and sporadic, potentially devastating pandemics. The humoral immune response plays an important role in the defense against these viruses, providing protection mainly by producing antibodies directed against the hemagglutinin (HA) glycoprotein. However, their high genetic variability allows the virus to evade the host immune response and the potential protection offered by seasonal vaccines. The emergence of resistance to antiviral drugs in recent years further limits the options available for the control of influenza. The development of alternative strategies for influenza prophylaxis and therapy is therefore urgently needed. In this study, we describe a human monoclonal antibody (PN-SIA49) that recognizes a highly conserved epitope located on the stem region of the HA and able to neutralize a broad spectrum of influenza viruses belonging to different subtypes (H1, H2 and H5). Furthermore, we describe its protective activity in mice after lethal challenge with H1N1 and H5N1 viruses suggesting a potential application in the treatment of influenza virus infections.

## Introduction

Seasonal influenza causes up to 500,000 deaths worldwide each year [1]. Infants, immunocompromised individuals and the elderly are particularly susceptible, with 90% of deaths occurring in the latter group [2]. Influenza viruses can also cause pandemics that, although rare, are recurrent events historically associated with high levels of morbidity and mortality [3], [4], [5], [6]. Preventive vaccination has historically been the most efficient measure of influenza control, but this approach presents important limitations due to the accumulation of antigenic mutations in the virus, known as antigenic drift. Vaccines typically elicit a potent neutralizing antibody response limited to the specific viral strains included in the preparation and to closely related viruses [2]. For this reason, seasonal vaccines need to be annually reformulated based upon the forecasting of viral strains that will circulate in the coming influenza season. Furthermore, influenza vaccines have suboptimal immunogenicity and efficacy in the groups at highest risk of severe disease [7]. Moreover in the case of a pandemic, the use of vaccine is limited by the time required for its development and deployment [8].

The current therapeutic regimen for influenza A viruses is limited to two classes of drugs: the adamantanes (amantadine and rimantadine) and the neuraminidase inhibitors (oseltamivir and zanamivir). However, the natural and/or acquired resistance to these drugs has been reported [9], [10]. Resistance to adamantanes is prevalent among seasonal and avian influenza A viruses significantly reducing their usefulness [11], [12]. The sudden and widespread emergence of resistance to oseltamivir among pre-pandemic H1N1 viruses has raised further concerns over the current therapeutic options [13], [14]. Oseltamivir resistance was reported in patients infected with the pandemic H1N1 viruses and highly virulent H5N1 viruses [15], [16]. The resistance to zanamivir is rare [17], but its use is limited to patients who can actively inhale it, which often excludes young children, impaired older adults or patients with underlying airway disease [14], that is the groups of patients most vulnerable to serious influenza infection complications.

Alternative strategies are needed to combat the constant threats posed by influenza. One of such strategies may come from passive immunoprophylaxis with monoclonal antibodies (mAbs) recognizing broadly conserved influenza epitopes and endowed with broad-range neutralizing activity [18].

The most important protective antigen on the surface of influenza virus is HA, whose structure can be divided in two distinct regions: the globular head, responsible for the binding to the sialic acid, and the stem region that contains the fusion peptide and the membrane anchor domain. On the globular head, constituted by the HA1 subunit, lie several epitopes targeted by neutralizing antibodies [18]. However, mAbs recognizing this region are of restricted application due to the antigenic drift that this region encounters [18]. In contrast, the stem region of HA, formed mostly by the HA2 subunit, is relatively conserved among different influenza A subtypes [19] and indeed could represent an universal target for the development of cross-neutralizing monoclonal antibodies.

Several human heterosubtypic neutralizing mAbs, directed against HA stem region and with protective features in animal models, have been recently described [20], [21], [22], [23], [24], [25] . All these mAbs recognize epitopes located in the most conserved region of influenza viruses HA and neutralize influenza viruses by blocking fusion of the viral and the host endosomal membranes. Many of these anti-influenza heterosubtypic neutralizing mAbs utilize the VH1-69 germline gene and bind to a hydrophobic region on the HA stem using their complementary determining region 2 (CDR2).

In this study, we describe a human monoclonal antibody named PN-SIA49 recognizing a highly conserved epitope on the stem region of HA and featuring one of the strongest in-vitro neutralizing activity described so far against a broad spectrum of viruses belonging to different influenza subtypes (H1, H2 and H5). Furthermore, we describe its protective activity in mice after lethal challenges with H1N1 and H5N1 viruses suggesting its potential as a broad-spectrum monoclonal antibody for treatment of influenza virus infection.

## Results

### Heterosubtypic neutralization of influenza virus subtypes by PN-SIA49

It was previously described that the Fab fragment of PN-SIA49 binds to the stem region of HA and neutralizes all tested H1N1 isolates [26], [27]. The heavy chain variable region of PN-SIA49 uses the VH3-23 gene and is paired with a VL1-39 light chain. The nucleotide sequence homology of PN-SIA49 with the germline sequence is 93.11% for the VH gene and 92.30% for the VL gene, demonstrating its origin from a somatic mutation process.

In this study, in order to better characterize its neutralizing activity, PN-SIA49 was produced as whole IgG1 molecule using the BD BaculoGold System. The whole IgG molecule was tested in fluorescence inhibition assay, infectious foci formation reduction assay and plaque reduction assay against human, swine and avian influenza A viruses belonging to phylogenetic group 1 (H1N1, H2N2, H5N1 and H9N2) and group 2 (H3N2 and H7N2). The results obtained showed that IgG PN-SIA49 has a stronger neutralizing activity compared to Fab PN-SIA49 (Table 1) [26], [27]. Indeed, IgG PN-SIA49 neutralized all tested viruses belonging to group 1, except the virus strain belonging to H9N2 subtype, with a half maximal inhibitory concentration (IC50) ranging between 0.1–1.9 µg/ml (Table 1 and Figure S1). On the contrary, PN-SIA49 showed no neutralizing activity against the viruses belonging to group 2 (Figure S2). These data suggest that the epitope recognized by PN-SIA49 is conserved among group 1 viruses.

**Table 1:** PN-SIA49 neutralizing activity against all tested influenza strains.

Subtype	Strain	Fab PN-SIA49	IgG PN-SIA49
		IC50^a^ µg/ml	IC50a µg/ml
H1N1	A/Wilson Smith/1933	4.5	0.5
	A/Puerto Rico/8/1934	2.1	0.4
	A/Malaya/302/1954	2.7	0.2
	A/Milan/UHSR1/2009	2.8	0.1
	A/swine/Parma/1/1997	2.3	1.1
H2N2	A/Ann Arbor/6/1960	ND^b^	1.1
H5N1	A/Vietnam/1203/2004 clade 1	ND	1.1
	A/duck/Vietnam/NCVD98/2007 clade 2.3.4	ND	1.9
H9N2	LAIV A/chicken/Hong Kong/G9/1997	ND	>10
H3N2	A/Aichi/2/1968	ND	>20
	A/Hong Kong/8/1968	ND	>30
	A/Port Chalmers/1/1973	ND	>20
	A/Philippines/01/1982	ND	>10
	A/England/648/1989	ND	>10
	A/Fukui/20/2004	ND	>10
	A/Washington/01/2007	ND	>30
	A/New Hampshire/01/2009	ND	>30
H7N2	A/New York/107/2003	ND	>10

a IC50: Half maximal inhibitory concentration expressed in µg/ml.

b ND: not determined.

### Therapeutic efficacy of PN-SIA49 against H1N1 and H5N1 challenge in a mouse model

**Figure 1:**
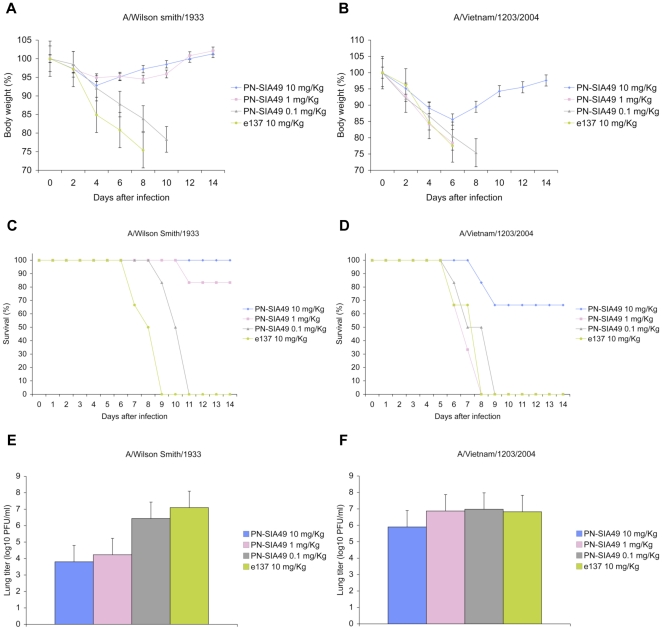
PN-SIA49 confers protection in mice against lethal challenge with both A/Wilson Smith/33 and A/Vietnam/1203/2004. Female BALB/c mice were challenged with 3 LD50 of A/Wilson Smith/33 (WS33) or A/Vietnam/1203/2004 (VN04). Twenty-four hours after the viral challenge, graded doses (10, 1, 0.1 mg/Kg) of PN-SIA49 or the control antibody (e137, 10 mg/Kg) were administrated to mice. Mice were monitored for body weight loss and survival for 2 weeks after challenge. (A) Body weight following WS33 virus challenge and (B) VN04 and treated with PN-SIA49. (C) Survival rates of mice challenged with WS33 and (D) VN04 after PN-SIA49 treatment. (E) Viral titers in the lungs of mice challenged with WS33 and (F) VN04 on day 4 post infection. Each data point represents the average viral titer from 4 mice. A group of mice treated with a negative control mAb (e137) was used and is included in each panel for direct comparison.

To determine whether the in-vitro neutralizing activity displayed by PN-SIA49 would be predictive of its protective efficacy in-vivo, BALB/c mice were inoculated intranasally with 3 fifty percent lethal dose (LD50) of A/Wilson Smith/33(H1N1) (WS33) or A/Vietnam/1203/2004 (H5N1) (VN04) virus, and were treated 24 hours later with 10, 1 or 0.1 mg/kg of PN-SIA49. An anti-HCV/E2 mAb (e137) [28] was used as control at 10 mg/kg. PN-SIA49 protected mice from lethal challenge with WS33 in a dose-dependent manner providing 100% protection (6/6) against death in animals that received 10 mg/kg of the antibody and 83.3% protection (5/6) in animals that received 1 mg/kg (Figure 1C). Consistent with the in-vitro neutralizing activity, PN-SIA49 at 10 mg/kg afforded 66.6% protection against lethal H5N1 virus challenge (Figure 1D) whereas control mice rapidly succumbed to infection by day 8 post-challenge (p.c.). Surviving mice remained healthy and showed minimal body weight loss (maximum weight loss: 7.2% in WS33 group, 14.4% in VN04 group) over the 2-week observation period. At the conclusion of the experiment, The mean body weight loss was 2.2% in the treated mice challenged with VN04 virus (Figure 1B) while mice infected with WS33 regained their full body weight (Figure 1A).

To gain further insights into the kinetics of viral neutralization in vivo, 4 mice from each group were euthanized on day 4 p.c. and viral titer was determined in whole lung tissues. PN-SIA49 significantly reduced virus titer in the lungs of mice infected with WS33 by approximately 2,000 fold at 10 mg/kg and about 700 fold at 1 mg/kg (Figure 1E). A 10-fold reduction in pulmonary virus titer was noted in mice challenged with VN04 virus that received PN-SIA49 at 10 mg/kg (Figure 1F).

Taken together, these results indicate that the survival was associated with an important reduction of the virus burden in the lungs of mice treated with PN-SIA49 and that the reduction is concordant with its in-vivo activity.

### Definition of the region bound by PN-SIA49 on HA

In order to better define the HA region recognized by PN-SIA49, several approaches were used.

Firstly, the hemagglutination inhibition (HI) activity for PN-SIA49 was evaluated and the resulting HI titre was 2.5 µg/ml and 0.039 µg/ml for VN04 and WS33 virus, respectively.

Secondly, to evaluate if PN-SIA49 recognizes an epitope on the HA stem region, a competition assay on A/Puerto Rico/8/1934-HA (A/PR/8/34-HA) human epithelial kidney HEK293T transfected cells was performed between PN-SIA49 and the commercially available mouse monoclonal antibody C179 (Takara Bio inc., Otsu, Shiga, Japan), which binds to an epitope on the HA stem region [29]. The results obtained showed that PN-SIA49 completely inhibited C179 binding to the A/PR/8/34-HA (Figure S3).

**Figure 2:**
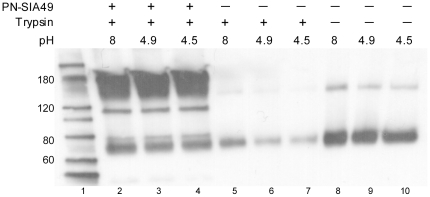
PN-SIA49 protects H1-HA from the low pH induced protease sensitivity. Western Blotting results of the protease susceptibility assay for H1-HA. Exposure to low pH converts HA to the protease-susceptible, post-fusion state (lane 6 and 7). Pre-treatment with PN-SIA49 partially blocks the pH-induced conformational change retaining HA in the protease-resistant, pre-fusion state (lane 3 and 4). In lanes 2–4 is also visible the signal given by PN-SIA49 (180 KDa). The other bands (about 110 KDa and 190 KDa) are due to HA aggregates bound by PN-SIA49 or by the primary anti-influenza Ab used in the western blotting assay.

Further evidence that PN-SIA49 binds to an epitope on the HA stem region is given by lack of protease susceptibility of the HA at low pH in the presence of PN-SIA49. Exposure to low pH followed by trypsin digestion results in degradation of HA. In contrast, when the HA is pre-treated with PN-SIA49, most HA is retained in a protease resistant, pre-fusion form (Figure 2).

Taken together, these preliminary data suggest that the epitope recognized by PN-SIA49 is localized in the HA stem region, but in close proximity of the HA globular head.

**Figure 3:**
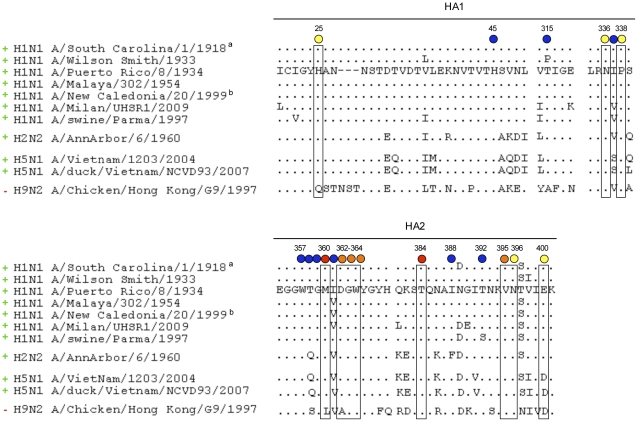
Amino acidic sequence conservation in hemagglutinin groups and subtypes at the region bound by PN-SIA49. Circles below residues indicate PN-SIA49 percentage binding to each HA alanine mutant compared to binding to the wild-type HA: red 25% binding; yellow 50–75% binding, blue 100% binding. Sequence numbering is based on H1N1 A/Puerto Rico/8/1934 coding region (GenBank accession number ABO21709). Subtypes that can be neutralized by PN-SIA49 are indicated with a green ‘+’ on the left, while the ones that can not be neutralized are indicate with a red ‘−’. ^a^ Recombinant HA from H1N1 A/South Carolina/1/1918 pandemic strain was previously shown to be bound by PN-SIA49 [26], [27]. ^b^ H1N1 A/New Caledonia/20/1999 was previously shown to be neutralized by PN-SIA49 as Fab fragment [26], [27].

Based on the PN-SIA49-C179 competition assay results, a large panel of HA mutants carrying an alanine substitution were generated [29], [30], [31], [32]. The binding of PN-SIA49 to these mutants was then evaluated by FACS analysis. Data obtained revealed that the binding of PN-SIA49 was decreased by His25Ala, Asn336Ala, Pro338Ala mutants on HA1 and Met360Ala, Asp362Ala, Gly363Ala, Trp364Ala, Thr384Ala, Val395Ala, Asn396Ala, Glu400Ala mutants located on HA2 (sequence numbering refers to A/PR/8/34, GenBank accession number ABO21709). Importantly, these residues are extremely conserved among viruses belonging to H1N1 subtype spanning from 1918 and 2009 and also highly conserved in H2N2 and H5N1 subtypes (Figure 3). All the other mutations did not have any effect on the antibody PN-SIA49 binding to the HA (Figure 3). To exclude the possiblity that the reduced PN-SIA49 binding to HA was due to reduced expression of HA on the cell surface, we performed a FACS analysis in which wild-type HA and mutants HA were stained with a mouse anti-influenza A HA (H1 subtype) monoclonal antibody. This antibody was directed against a linear epitope in order to evaluate the expression level for each HA on cell surface. As shown in figure S4, the HA mutants are expressed on the cell surface at a similar levels to that of wild type HA.

Consistent with the great phylogenetic distance, the amino acidic difference in positions 25, 360 and 362 between the tested viral strains belonging to H1N1, H2N2, H5N1 subtypes and the viral strains belonging to H9N2 subtype may at least partially explain the lack of neutralizing activity against the H9N2 strain.

**Figure 4:**
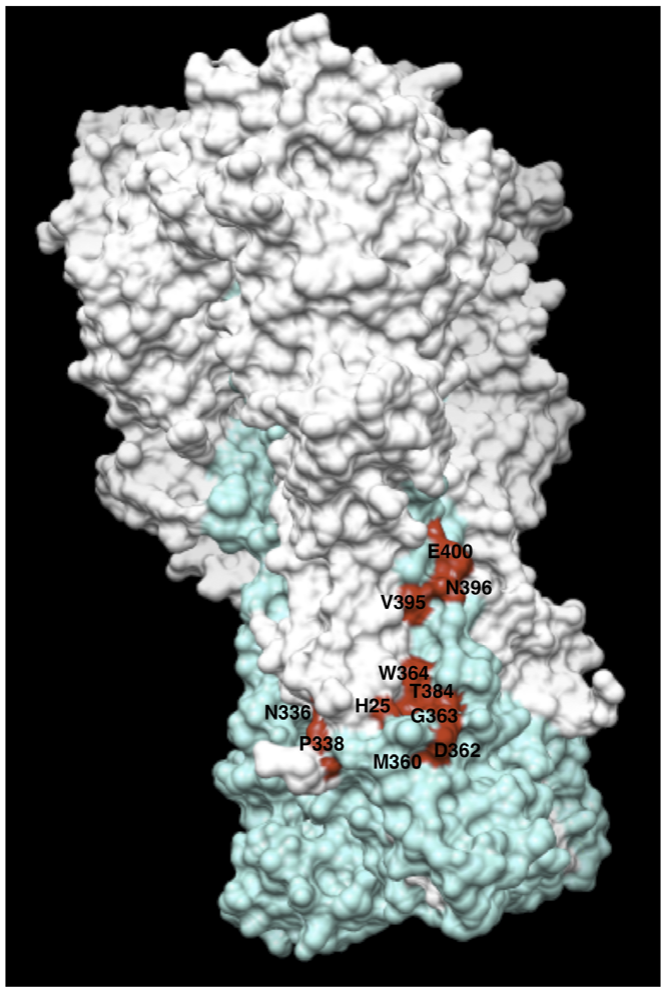
Amino acidic residues involved in the binding of PN-SIA49 to A/Puerto Rico/8/1934 (H1N1) hemagglutinin. The HA of A/Puerto Rico/8/1934 is represented as trimer with the HA1 domains colored white and the HA2 domains colored cyan. Mutations that affect the binding of PN-SIA49 are highlighted in red.

Based on the results obtained from the alanine scanning study, an in-silico analysis on the HA crystal structure of A/PR/8/34 (PDB ID code 1RU7) was carried out. The analysis confirmed that the residues identified lie on the stem region of HA, that they belong to the HA1 and HA2 subunits and that they are exposed on the surface of the HA molecule (Figure 4).

## Discussion

In this study, we characterize a human mAb, IgG PN-SIA49, directed against influenza viruses and previously described as Fab fragment [26], [27]. IgG PN-SIA49 neutralizes a broad spectrum of viruses belonging to different influenza subtypes (H1, H2 and H5) and is characterized by the lowest mean IC50 values described to date for a heterosubtypic mAb [20], [21], [22], [23], [24], [25] against the influenza subtypes tested in this study (Table S1). Furthermore, IgG PN-SIA49 is endowed with a stronger neutralizing activity compared to its monovalent molecule (Table 1). Indeed, it is well documented in the literature that bivalency of IgG molecule may be an essential feature for the biological activity of a mAb, mostly due to an increase in antibody avidity [33], [34], [35], [36]. The increased avidity may also play an important role in the prophylactic and therapeutic application of an antibody, allowing the administration of smaller amount compared to the Fab fragment. Additionally, the whole IgG molecule has a longer half-life than its fragments, with a consequent prolonged effect of the molecule. For all these reasons, we used IgG PN-SIA49 to treat mice infected with H1N1 and H5N1 viruses and data obtained show that PN-SIA49 protects mice from H1N1 and H5N1 virus lethal challenge suggesting its potential application in treatment of influenza virus infections.

Most of the anti-influenza heterosubtypic neutralizing mAbs described thus far, derived from the VH1-69 germline, bind to a conserved epitope in the HA stem region present only on group 1 influenza A viruses. The binding is mainly through highly hydrophobic amino acidic residues in the heavy chain CDR2 [20], [21], [22], [23], [24], [25]. Instead, the heavy chain variable region of PN-SIA49 results from VH3-23 gene rearrangement with the D3-3 and JH6 gene segments demonstrating that, despite the preferential usage of VH1-69 in the heterosubtypic response to influenza HA [20], an in-vivo heterosubtypic protection may be conferred also by non VH1-69 derived Abs.

PN-SIA49 recognizes a novel broadly neutralizing epitope on HA stem region, which is highly conserved among group 1 subtypes that have been confirmed in humans. In particular, this region is broadly shared among H1N1 isolates spanning from 1918 to 2009, H2N2 subtype responsible for the 1957 pandemic, and highly pathogenic and potentially pandemic H5N1 influenza virus.

Finally, PN-SIA49 is protective in mice when given after lethal challenge with either H1N1 or H5N1 virus. This suggests its great potential as broad-spectrum monoclonal antibody for in vivo treatment of influenza virus infections.

Taken together, these data underline the importance of PN-SIA49 for the development of anti-influenza strategies based on passive immunization. Further studies will be necessary to define the most effective prophylactic and therapeutic administration protocols. Finally, these data and a more detailed definition of the epitope recognized by PN-SIA49 could also be useful to develop new vaccination strategies able to elicit a humoral immune response directed against key regions of the influenza HA protein.

## Methods

### Ethics Statement

This study was carried out in strict accordance with the recommendations in the Guide for the Care and Use of Laboratory Animals, National Research Council (Eighth Edition, 2011). All mouse procedures were approved by Institutional Animal Care and Use Committee (IACUC) of the Centers for Disease Control and Prevention and performed in accordance with the IACUC guidelines (Protocol # 1619: “Studies on the Pathogenesis of and Immunity to Influenza Viruses in Mice”).

### Monoclonal antibodies

We previously described the molecular cloning of a human monoclonal antibody Fab fragment named PN-SIA49 [26], [37]. The whole antibody molecule was obtained using the BD BaculoGold System (BD Biosciences Pharmingen, San Diego, CA, USA). Briefly, nucleotide sequences codifying heavy and light chains of the PN-SIA49 Fab fragment were cloned into the baculovirus expression vector pAc-κ-Fc (PROGEN Biotechnik GmbH, Heidelberg, Germany). Sf9 insect cells (Invitrogen, Carlsbad, CA, USA) were co-transfected with the linearized baculovirus DNA (BD Biosciences Pharmingen, San Diego, CA, USA) and the pAc-κ-Fc/PN-SIA49. The obtained virus was inoculated at a multiplicity of infection (MOI) of 5 to infect 1×109 High Five insect cells (Invitrogen, Carlsbad, CA, USA) in a final volume of 1 liter. After an incubation of 96 hours the culture media was collected, clarified by centrifugation and filtered with 0.2 µm filter (Millipore, Billerica, MA, USA). The media was loaded into a protein G column (Amersham Biosciences GE Healthcare, Zurich, Switzerland), the antibody was eluted with citric acid 0.1 M, pH 3, and immediately neutralized with Tris Base 1 M, pH 9. The solution containing the antibody was dialyzed against PBS and then concentrated using Amicon Ultra-15 Centrifugal Filter Devices (Millipore, Billerica, MA, USA). Antibody concentration was determined by SDS-PAGE gel and by spectrophotometric measurement at 280 nm.

An anti-influenza A antibody directed against H1N1 subtype HA, named RB62, and an anti-HCV E2 glycoprotein antibody, named e137 [28], produced and purified with an identical procedure were used as controls in all experiments.

### Viruses and cells

The following human H1N1 and H3N2 reference strains were acquired from the American Type and Culture Collection (ATCC): A/Puerto Rico/8/1934 (H1N1) (ATCC n. VR-1469); A/Wilson Smith/1933 (H1N1) (ATCC n. VR-1520); A/Malaya/302/1954 (H1N1) (ATCC n. VR-98); A/Hong Kong/8/1968 (H3N2) (ATCC n. VR-544); A/Aichi/2/1968 (H3N2) (ATCC n. VR-547); A/Port Chalmers/1/1973 (H3N2) (ATCC n. VR-810). The swine origin influenza virus (S-OIV) A/Milan/UHSR1/2009 (H1N1v) was previously isolated in our laboratory [26], [37]. A swine strain, A/swine/Parma/1/1997 (H1N1), was kindly provided by the Zooprophylactic Institute of Brescia, Italy. All these viruses were tested in the BLS3 laboratory of the Vita-Salute San Raffaele University. The following H1N1, H2N2, H3N2, H5N1, H7N2 and H9N2 viruses were tested at the Centers for Disease Control and Prevention (CDC) of Atlanta, Georgia, USA: A/Wilson Smith/1933 (H1N1); A/Ann-Arbor/6/1960 (H2N2); A/Hong Kong/8/1968 (H3N2); A/Pilippines/01/1982 (H3N2); A/England/648/1989 (H3N2); A/Fukui/20/2004 (H3N2); A/Washington/01/2007 (H3N2); A/NewHampshire/01/2009 (H3N2); A/Vietnam/1203/2004 (H5N1) clade 1; A/duck/Vietnam/NCVD98/2007 (H5N1) clade 2.3.4; A/New York/107/2003 (H7N2); LAIV A/chicken/Hong Kong/G9/1997 (H9N2).

All viruses, excluding the A/swine/Parma/1/1997, were cultured on Madin-Darby Canine Kidney (MDCK) (ATCC CCL-34) cells propagated in Modified Eagle Medium (MEM) (Gibco Invitrogen, Carlsbad, CA, USA) supplemented with 2% bovine serum albumin (Gibco Invitrogen, Carlsbad, CA, USA), 50 µg/ml of penicillin (Gibco Invitrogen, Carlsbad, CA, USA), 100 µg/ml of streptomycin (Gibco Invitrogen, Carlsbad, CA, USA) and 2 µg/ml TPCK–trypsin (Roche Applied Science). The A/swine/Parma/1/97 isolate was analogously grown on Newborn Swine Kidney (NSK) cells, kindly provided by the Zooprophylactic Institute of Brescia, Italy. At 80% confluence, cells in MEM supplemented with 2 µg/ml serum-free TPCK-trypsin, were infected with each strain at a MOI of 0.001. After 1 hour of infection, cells were washed with phosphate buffered saline (PBS); MEM supplemented with 2 µg/ml TPCK-trypsin was then added and cells were incubated at 37°C in 5% CO2 atmosphere. Cells were observed daily to monitor the cytopathic effect and, usually after 72–96 hours, the supernatant was collected, centrifuged at 1000 rcf for 10 minutes to eliminate cells debris and filtered with 0.22 µm filters (Millipore, Billerica, MA, USA). The supernatant was then aliquoted and stored at −80°C as cell-free virus.

### Virus neutralization assays

#### Fluorescence inhibition assay and plaque reduction assay

The following H1N1 and H3N2 viruses were tested using the fluorescence inhibition assay: A/Puerto Rico/8/1934 (H1N1); A/Wilson Smith/1933 (H1N1); A/Malaya/302/1954 (H1N1); A/Milan/UHSR1/2009 (H1N1v); A/swine/Parma/1/1997 (H1N1); A/Hong Kong/8/1968 (H3N2); A/Aichi/2/1968 (H3N2); A/Port Chalmers/1/1973 (H3N2). Each viral isolate was titrated by the limiting dilution method and the viral titer calculated by the Reed-Muench formula. Neutralizing assays were carried out in 96 wells plate using MDCK cells (4×104cells/well). Serial dilutions, 10 µg/ml-0.03 µg/ml, of IgG PN-SIA49 were preincubated for 1 hour at 37°C with 100 median tissue culture infective doses (TCID50) of virus. Following incubation, 100 µl of the mix antibody-virus were added to the cells and incubated for another hour at 37°C in 5% CO2. At the end of this incubation, cells were washed with PBS and 100 µl of MEM TPCK-Trypsin (2 µg/ml) were added in each well. Cells were incubated for 7 hours at 37°C in 5% CO2 and then washed with PBS, fixed and permeabilized with ice-cold ethanol. Cells were incubated with anti-influenza A mouse antibody (Argene, Shirley, NY, USA) for 30 minutes at 37°C in a humid chamber. The cells were then washed with PBS and incubated for 30 minutes at 37°C in a dark humid chamber with a FITC-conjugated secondary antibody (Argene, Shirley, NY, USA). Nuclei staining was obtained with Hoechst 33342 (Sigma Aldrich). An infection control without antibody was included, as well as a negative control with the anti-HCV/E2 antibody e137. Each neutralization assay was performed in triplicate and repeated in two different sessions.

The neutralization activity for each antibody concentration was expressed as the percentage reduction of fluorescent nuclei compared with the nuclei count in the infection control. Nuclei counting was performed by using the GE Healthcare's IN Cell Analyzer 1000, an automated epifluorescence based microscope system. The neutralization curves were then fit by non-linear regression with the GraphPad Prism software, allowing IC50 calculation.

The A/PR/8/34 (H1N1) and A/Milan/UHSR1/2009 (H1N1) viruses were also tested in plaque reduction assay as previously described [26]. Briefly, neutralizing assays were carried out in 6 wells plates using MDCK cells (5×105 cells/well). Two dilutions, 1-0.1 µg/ml, of IgG PN-SIA49 were preincubated for 1 hour at 37°C with 100 TCID50 of virus. Following this incubation, 1 ml of each virus-antibody mix was added on MDCK monolayer and the plate was incubated 1 hour at 37°C in 5% CO2. Then, the medium was removed and the monolayer washed twice with PBS. Two ml of MEM-agarose 0.8% supplemented with penicillin (50 µg/ml) (Gibco Invitrogen, Carlsbad, CA, USA), streptomycin (100 µg/ml) (Gibco Invitrogen, Carlsbad, CA, USA), L-glutamine (2 mM) (Gibco Invitrogen, Carlsbad, CA, USA) and trypsin (2 µg/ml) (Roche Applied Sciences) were added to each well and the plates were incubated 48 hours at 37°C in 5% CO2. After this incubation, the agarose medium was removed from each well and 1 ml of 70% methanol-crystal violet 1% (w/v) was added to each well at room temperature. Finally, the wells were washed with tap water and dried. An infection control without antibody was added as well as a negative control with anti-HCV/E2 e137 mAb. The neutralization was determined counting the PFU reduction in presence of antibodies compared to the infection control.

#### Infectious foci formation reduction assay

The following H1N1, H2N2, H3N2, H5N1, H7N2 and H9N2 viruses were tested: A/Wilson Smith/1933 (H1N1); A/Ann-Arbor/6/1960 (H2N2); A/Hong Kong/8/1968 (H3N2); A/Pilippines/01/1982 (H3N2); A/England/648/1989 (H3N2); A/Fukui/20/2004 (H3N2); A/Washington/01/2007 (H3N2); A/NewHampshire/01/2009 (H3N2); A/Vietnam/1203/2004 (H5N1) clade 1; A/duck/Vietnam/NCVD98/2007 (H5N1) clade 2.3.4; A/New York/107/2003 (H7N2); LAIV A/chicken/Hong Kong/G9/1997 (H9N2). Each viral isolate was titrated to establish working dilution that produces 15-30 foci forming units per well in 96 tissue culture plates. Neutralizing assays were carried out in 96 wells plate using MDCK/SIAT-1 cells. Serial dilutions, 30 µg/ml-0.37 µg/ml, of IgG PN-SIA49 were preincubated for 1 hour at 37°C with the subset of viruses. Following this incubation, 100 µl of the antibody-virus mix was added to the cells and incubated for another hour at 37°C in 5% CO2. At the end of this incubation, the cells were washed twice in PBS and 100 µl of virus growth media containing 2 µg/ml of TPCK treated trypsin was added. Cells were incubated for 12–16 hours at 37°C in 5% CO2 and then washed with PBS, fixed and permeabilized with ice cold methanol/acetic acid (95∶5) for 30 min at −20°C. Cells were incubated with anti-NP antibodies (Millipore, Billerica, MA, USA) for 30 minutes at 37°. The cells were then washed and incubated for 30 minutes at 37°C with a mouse HRP-conjugated secondary antibody. True Blue chromogenic substrate (KPL) was used to count the number of foci.

### Murine lethal challenge models

Female BALB/c mice were purchased at 6 to 8 weeks of age from Charles River Co. (Wilmington, MA). All mice were maintained in specific pathogen–free barrier facilities. All animal experiments and procedures conformed to protocols approved by the Centers for Diseases Control and Prevention (CDC), Atlanta, GA, USA.

For each virus, four groups of 10 mice were inoculated intranasally with 3 LD50 of A/Wilson Smith/33 or A/Vietnam/1203/2004 virus in a 50 µl volume. At 24 h after inoculation, graded doses (10, 1, 0.1 mg/Kg) of PN-SIA49 or the control antibody (e137, 10 mg/Kg) were administrated to mice by intraperitoneal injection in a final volume of 0.2 ml. A subset of six mice in each group were weighed on the day of virus challenge and then observed and weighed every 2 days for 2 weeks after inoculation. Mice that lost more than 25% of their initial body weight were euthanized.

A subset of four animals treated with mAbs were euthanized on day 4 after inoculation, and whole lungs were homogenized in 1 ml of sterile PBS. Virus titers in lung tissue homogenates were determined by plaque titration in MDCK cell monolayer cultures.

### Definition of the region bound by PN-SIA49 on HA

#### Hemagglutination (HI) assay

HI tests using mAb PN-SIA49 or e137 control antibody against live WS33 and VN04 viruses were performed according to standard protocols [38]. Briefly, serial dilutions of purified mAbs in PBS were performed from initial concentration of 5 µg/mL. Positive and negative control ferret sera were diluted initially 1∶10 in receptor-destroying enzyme from Vibrio cholerae (Denka Seiken, Tokyo). Serial dilutions of control sera or mAbs were pre-incubated with 4 HA units of virus per well. For WS33 virus, turkey red blood cells (RBCs) were added to a final concentration of 0.5%, whereas horse RBCs were used at a 1% suspension for VN04 virus. Normal ferret serum gave a value of less than 10. Specific HI activity of mAbs was calculated as the lowest concentration of mAb that displayed HI activity.

#### Protease susceptibility assay

Each reaction contained 3 µl of the anti-influenza vaccine season 2011–2012 (InflexalV-Crucell), which contains 30 ng of A/California/7/09 (H1N1) HA or 3 µl of the anti-influenza vaccine season 2011–2012 combined with 2 fold molar excess of PN-SIA49. Titron X-100 was added to prevent aggregation of the post-fusion HA. The pH was lowered in all samples except controls using citric acid 0.1 M pH 3. Reactions were mixed, briefly centrifuged and incubated at 37°C for one hour. After incubation, reactions were equilibrated to room temperature and the pH was neutralized by addition of 1 M Tris, pH 9. The actual pH reached was determined in parallel using larger buffer volumes without protein. Trypsin was added to all samples except controls at a final ratio of 1∶25 by mass and samples were digested overnight at 37°C. Non-reducing SDS buffer was added to each reaction. Samples were boiled for ∼2 minutes and loaded on a non-reducing 4–15% polyacrilamide pre-casted gel (Biorad, Italy). After running, samples were transferred on a PVDF membrane (PerkinElmer, Belgium) for 2 hours at 350 mA. The membrane was then blocked with 5% not fat milk in PBS-Tween20 0,1% (PBST) for 1 h at room temperature and then washed three times with PBST. PN-SIA28, a human anti-HA monoclonal antibody recognizing HA0 [39], was used as primary antibody at 1 µg/ml in 5% not fat milk-PBST. The membrane was incubated for 1 hour at room temperature and then was washed three times with PBST. Secondary anti-human antibody was added and incubated for 1 h at room temperature. After incubation the membrane was washed, the substrate solution (SuperSignal® West Pico Chemiluminescent Substrate, PIERCE) was added and incubated for 2 min.

To determine the pH required to convert the HA to the post-fusion form, pH titrations using the assay describing above was performed. Samples were exposed to a range of pH conditions (pH 4.5, 4.9, 5.3, 5.7, 6.1, 6.5, 7 and 8), neutralized and processed as described above.

### Hemagglutinin cloning and mutagenesis

A/Puerto Rico/8/1934 (H1N1) hemagglutinin (A/PR/8/34-HA) was amplified as previously described [26], [37] using the following PCR primers:

APR834_fw:CACCATGAAGGCAAACCTACTGGTCCTGTTATGTG.

APR834_rev:TCAGATGCATATTCTGCACTGCAAAGATCCATTAGA.

The PCR products were cloned into the pcDNA 3.1D/V5-His-TOPO vector (Invitrogen, Carlsbad, CA, USA). Subsequently, HA alanine mutants were generated using Gene Tailor Site-Directed Mutagenesis System (Invitrogen, Carlsbad, CA, USA). A total of 20 A/PR/8/34-HA mutants were generated (His25Ala, His45Ala, Thr315Ala, Asn336Ala, Ile337Ala, Pro338Ala on the HA1 subunit and Trp357, Thr358, Gly359, Met360, Ile361, Asp362, Gly363, Trp364, Thr384, Ile388, Thr392, Val395, Asn396, Glu400 on the HA2 subunit. Sequence numbering refers to A/PR/8/34, GenBank accession number ABO21709).

### Cytofluorimetric binding assays

The binding activity of PN-SIA49 was assayed using full-length wild type and mutants HAs. Human epithelial kidney HEK293T cells (ATCC CRL-1573) were transfected in 6 wells plate (Corning, Corning, NY, USA) (1×106 cells/well) with 4 µg of pcDNA 3.1D/V5-His-TOPO vector containing the HA nucleotide sequences described above. After centrifugation and fixation with 4% paraformaldehyde for 15 minutes at RT, the transfected cells were incubated for 30 minutes at room temperature with PN-SIA49 or conformational controls for H1N1 (RB62) at 10 µg/ml. Additionally, the isotype control, e137 (10 µg/ml) was introduced as well as untransfected cells and a mouse anti-influenza A HA (H1 subtype) monoclonal antibody (GeneTex Inc., Irvine, CA, USA) directed against a linear epitope to evaluate the transfection efficiency and the expression level for each HA. The cells were then washed with PBS and incubated for 30 minutes at room temperature with FITC-conjugated anti-human (Sigma Aldrich) or anti-mouse (Argene, Shirley, NY, USA) antibody. Afterwards, the cells were washed with PBS and analyzed by FACS. The FACS data were analyzed using the software Weasel w 2.5 (Waler+Eliza Hall, Institute of Medical Research, Parkville Victoria, Australia). The binding of PN-SIA49 to the different HA-mutants was then expressed as a binding percentage compared to wild-type. The data showing the PN-SIA 49 binding decrease between H1N1 wild type HA and H1N1 HA mutants, were obtained normalizing each PN-SIA 49 binding value to corresponding anti-H1 expression control values.

For the competition assay, serial dilutions of PN-SIA49 were used in combination with a fixed concentration (1 µg/ml) of mouse monoclonal antibody C179 (Takara Bio inc., Otsu, Shiga, Japan) which binds to an epitope on the HA stem region [29].

### Software

For sequences analysis the following software packages were used: SeqScape (Applied Biosystems), ClustalX (Toby Gibson), Bio Edit (Tom Hall, Ibis Therapeutics) and Treeview (GubuSoft). For molecular visualization and rendering UCSF Chimera package from the Resource for Biocomputing Visualization and Informatics at University of California, RasMol (Roger Sayle), Jmol (Jmol: an open-source Java viewer for chemical structures in 3D. http://www.jmol.org/), Cn3D (United States National Library of Medicine, NLM) were used. Finally for data analysis and graphical editing GraphPad Prism was used.

## Supporting Information

Figure S1 **Neutralization assays against group 1 influenza viruses.** Dose-response curve fit nonlinear regression is reported for IgG PN-SIA49 against neutralized group 1 influenza viruses. (A) Results from fluorescence inhibition assays, (B) plaque reduction assays and (C) infectious foci formation reduction assays. Data from at least two different experiments for each virus are reported. Each point was performed in triplicate.
(PDF)

Figure S2 **Influenza hemagglutinin unrooted phylogenetic tree of all the viral strains tested in neutralization assays with PN-SIA49.** Viral isolates belonging to group 1 and group 2 are divided into two different boxes. Subtypes that can be neutralized by PN-SIA49 are indicated with a green ‘+’, while the ones that cannot be neutralized are indicate with a red ‘−’. As reported in the text, PN-SIA49 is able to neutralize all of the group 1 viruses tested in this study except for the H9N2 strain. No neutralizing activity was detected against the H3N2 viruses tested. * The recombinant HA from A/South Carolina/1/1918 (H1N1) pandemic strain was previously shown to be bound by PN-SIA49 [26], [27]. # H1N1 A/New Caledonia/20/1999 was previously shown to be neutralized by PN-SIA28 as Fab fragment [26], [27].
(PDF)

Figure S3 **C179/PN-SIA49 competition assay.** Graphic representation of cell staining and flow cytometric analysis of HEK293T cells transfected with the pcDNA 3.1D/V5-His-TOPO vector containing the HA-A/PR/8/34 were performed. Serial dilutions of PN-SIA49 were used in combination with a fixed concentration (1 µg/ml) of C179 (blue line). A monoclonal antibody directed against the HA globular head was used as competition negative control (pink line).
(PDF)

Figure S4 **HA mutants that determine a decrease of PN-SIA49 binding to HA are expressed at the same level of wild type HA on cell surface.** FACS curves showing the binding of anti-H1N1 HA antibody (directed against a linear epitope) to untransfected cells, HA wild-type and HA-mutants. White and red curves represent, for each graph, respectively the binding of anti-HA expression control to untransfected cells and wild type H1N1-HA. The different colour curves represent the different mutants.
(PDF)

Table S1 Major anti-influenza human monoclonal antibodies with heterosubtypic neutralizing activity.
(DOC)

## References

[1] Thompson WW, Shay DK, Weintraub E, Brammer L, Bridges CB (2004). Influenza-associated hospitalizations in the United States.. Jama.

[2] Fiore AE, Shay DK, Broder K, Iskander JK, Uyeki TM (2008). Prevention and control of influenza: recommendations of the Advisory Committee on Immunization Practices (ACIP), 2008.. MMWR Recomm Rep.

[3] Fraser C, Donnelly CA, Cauchemez S, Hanage WP, Van Kerkhove MD (2009). Pandemic potential of a strain of influenza A (H1N1): early findings.. Science.

[4] Palese P (2004). Influenza: old and new threats.. Nat Med.

[5] Trifonov V, Khiabanian H, Rabadan R (2009). Geographic dependence, surveillance, and origins of the 2009 influenza A (H1N1) virus.. N Engl J Med.

[6] Webster RG (1999). 1918 Spanish influenza: the secrets remain elusive.. Proc Natl Acad Sci U S A.

[7] Smith NM, Bresee JS, Shay DK, Uyeki TM, Cox NJ (2006). Prevention and Control of Influenza: recommendations of the Advisory Committee on Immunization Practices (ACIP).. MMWR Recomm Rep.

[8] Monto AS, Ansaldi F, Aspinall R, McElhaney JE, Montano LF (2009). Influenza control in the 21st century: Optimizing protection of older adults.. Vaccine.

[9] Cheng PK, Leung TW, Ho EC, Leung PC, Ng AY (2009). Oseltamivir- and amantadine-resistant influenza viruses A (H1N1).. Emerg Infect Dis.

[10] Ramirez-Gonzalez JE, Gonzalez-Duran E, Alcantara-Perez P, Wong-Arambula C, Olivera-Diaz H (2011). Oseltamivir-Resistant Pandemic (H1N1) 2009 Virus, Mexico.. Emerg Infect Dis.

[11] Bright RA, Shay DK, Shu B, Cox NJ, Klimov AI (2006). Adamantane resistance among influenza A viruses isolated early during the 2005–2006 influenza season in the United States.. Jama.

[12] He G, Qiao J, Dong C, He C, Zhao L (2008). Amantadine-resistance among H5N1 avian influenza viruses isolated in Northern China.. Antiviral Res.

[13] Enserink M (2009). Drug resistance. A ‘wimpy’ flu strain mysteriously turns scary.. Science.

[14] Moscona A (2009). Global transmission of oseltamivir-resistant influenza.. N Engl J Med.

[15] Collins PJ, Haire LF, Lin YP, Liu J, Russell RJ (2008). Crystal structures of oseltamivir-resistant influenza virus neuraminidase mutants.. Nature.

[16] de Jong MD, Tran TT, Truong HK, Vo MH, Smith GJ (2005). Oseltamivir resistance during treatment of influenza A (H5N1) infection.. N Engl J Med.

[17] Moscona A (2008). Medical management of influenza infection.. Annu Rev Med.

[18] Hanson BJ, Boon AC, Lim AP, Webb A, Ooi EE (2006). Passive immunoprophylaxis and therapy with humanized monoclonal antibody specific for influenza A H5 hemagglutinin in mice.. Respir Res.

[19] Krystal M, Elliott RM, Benz EW, Young JF, Palese P (1982). Evolution of influenza A and B viruses: conservation of structural features in the hemagglutinin genes.. Proc Natl Acad Sci U S A.

[20] Corti D, Suguitan AL, Pinna D, Silacci C, Fernandez-Rodriguez BM (2010). Heterosubtypic neutralizing antibodies are produced by individuals immunized with a seasonal influenza vaccine.. J Clin Invest.

[21] Ekiert DC, Friesen RH, Bhabha G, Kwaks T, Jongeneelen M (2011). A highly conserved neutralizing epitope on group 2 influenza A viruses.. Science.

[22] Friesen RH, Koudstaal W, Koldijk MH, Weverling GJ, Brakenhoff JP (2010). New class of monoclonal antibodies against severe influenza: prophylactic and therapeutic efficacy in ferrets.. PLoS One.

[23] Kashyap AK, Steel J, Rubrum A, Estelles A, Briante R (2010). Protection from the 2009 H1N1 pandemic influenza by an antibody from combinatorial survivor-based libraries.. PLoS Pathog.

[24] Sui J, Hwang WC, Perez S, Wei G, Aird D (2009). Structural and functional bases for broad-spectrum neutralization of avian and human influenza A viruses.. Nat Struct Mol Biol.

[25] Throsby M, van den Brink E, Jongeneelen M, Poon LL, Alard P (2008). Heterosubtypic neutralizing monoclonal antibodies cross-protective against H5N1 and H1N1 recovered from human IgM+ memory B cells.. PLoS One.

[26] Burioni R, Canducci F, Mancini N, Clementi N, Sassi M (2010). Monoclonal antibodies isolated from human B cells neutralize a broad range of H1 subtype influenza A viruses including swine-origin Influenza virus (S-OIV).. Virology.

[27] Burioni R, Canducci F, Mancini N, Clementi N, Sassi M (2009). Molecular cloning of the first human monoclonal antibodies neutralizing with high potency swine-origin influenza A pandemic virus (S-OIV).. New Microbiol.

[28] Perotti M, Mancini N, Diotti RA, Tarr AW, Ball JK (2008). Identification of a broadly cross-reacting and neutralizing human monoclonal antibody directed against the hepatitis C virus E2 protein.. J Virol.

[29] Okuno Y, Isegawa Y, Sasao F, Ueda S (1993). A common neutralizing epitope conserved between the hemagglutinins of influenza A virus H1 and H2 strains.. J Virol.

[30] Cunningham BC, Wells JA (1989). High-resolution epitope mapping of hGH-receptor interactions by alanine-scanning mutagenesis.. Science.

[31] Matthews BW (1996). Structural and genetic analysis of the folding and function of T4 lysozyme.. FASEB J.

[32] Weiss GA, Watanabe CK, Zhong A, Goddard A, Sidhu SS (2000). Rapid mapping of protein functional epitopes by combinatorial alanine scanning.. Proc Natl Acad Sci U S A.

[33] Lamarre A, Talbot PJ (1995). Protection from lethal coronavirus infection by immunoglobulin fragments.. J Immunol.

[34] Ma JK, Hunjan M, Smith R, Kelly C, Lehner T (1990). An investigation into the mechanism of protection by local passive immunization with monoclonal antibodies against Streptococcus mutans.. Infect Immun.

[35] Yoden S, Kida H, Yanagawa R (1985). Is bivalent binding of monoclonal antibodies to different antigenic areas on the hemagglutinin of influenza virus required for neutralization of viral infectivity?. Arch Virol.

[36] Zhang MY, Xiao X, Sidorov IA, Choudhry V, Cham F (2004). Identification and characterization of a new cross-reactive human immunodeficiency virus type 1-neutralizing human monoclonal antibody.. J Virol.

[37] Burioni R, Canducci F, Clementi M (2009). Pregnancy and H1N1 infection.. Lancet.

[38] World Health Organization (2002).

[39] Clementi N, De Marco D, Mancini N, Solforosi L, Moreno GJ (2011). A Human Monoclonal Antibody with Neutralizing Activity against Highly Divergent Influenza Subtypes.. PloS one.

