# Supplementary figures and images for: A Non-VH1-69 Heterosubtypic Neutralizing Human Monoclonal Antibody Protects Mice against H1N1 and H5N1 Viruses
Source: PLoS One. 2012 Apr 4;7(4):e34415. doi: 10.1371/journal.pone.0034415 (PMC3319592; doi:10.1371/journal.pone.0034415)

**A**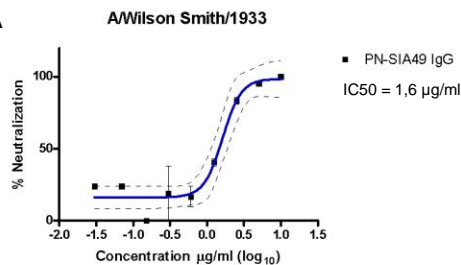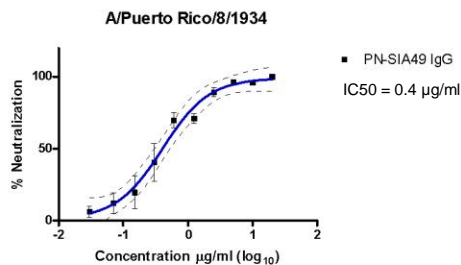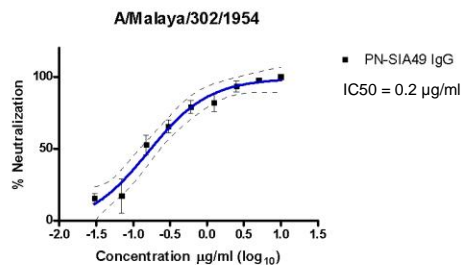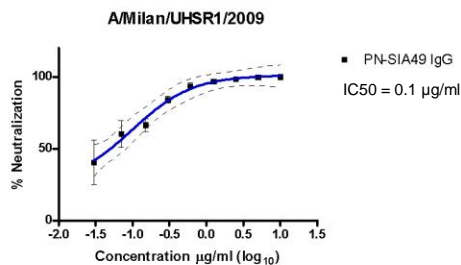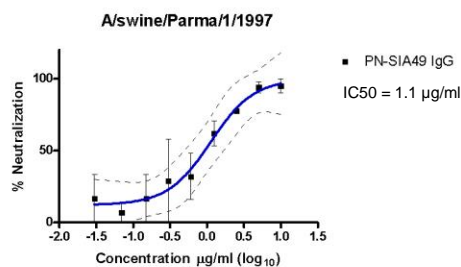**B**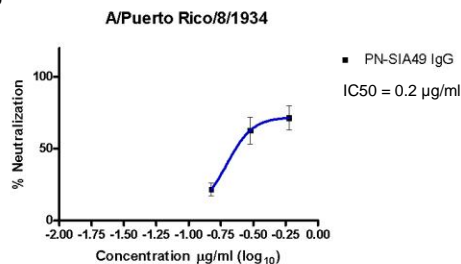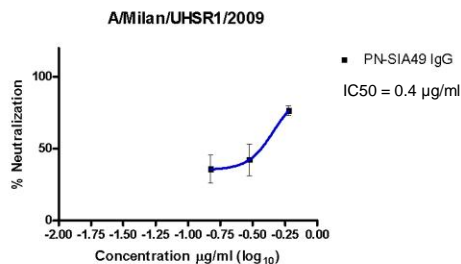**C**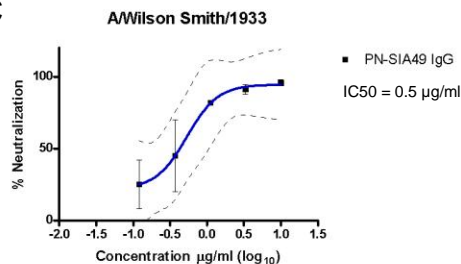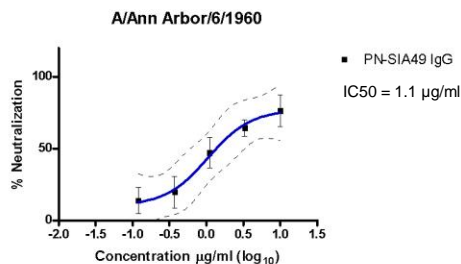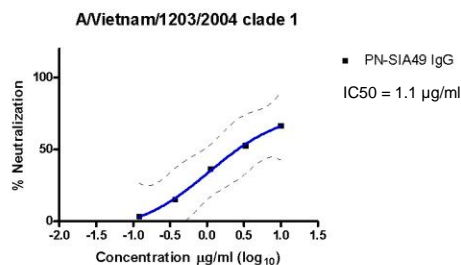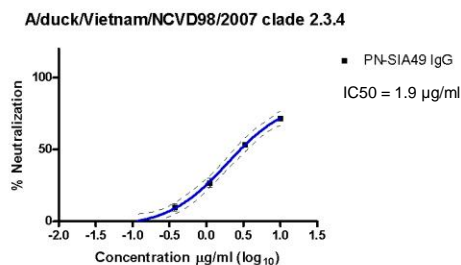

Supplement: Figure S1 — Neutralization assays against group 1 influenza viruses. Dose-response curve fit nonlinear regression is reported for IgG PN-SIA49 against neutralized group 1 influenza viruses. (A) Results from fluorescence inhibition assays, (B) plaque reduction assays and (C) infectious foci formation reduction assays. Data from at least two different experiments for each virus are reported. Each point was performed in triplicate. (PDF) [file pone.0034415.s001.pdf]

## Group 1

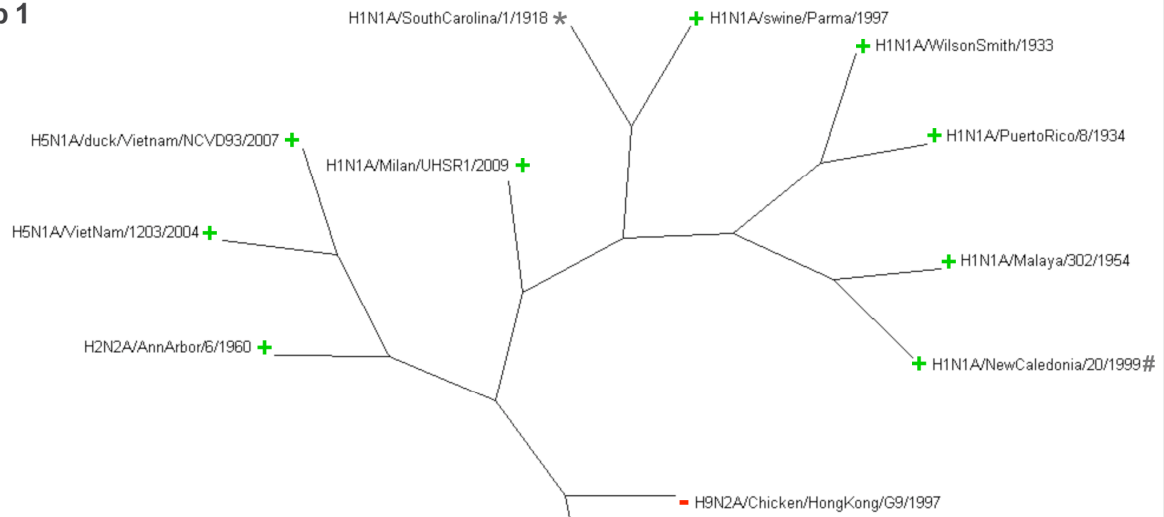

## Group 2

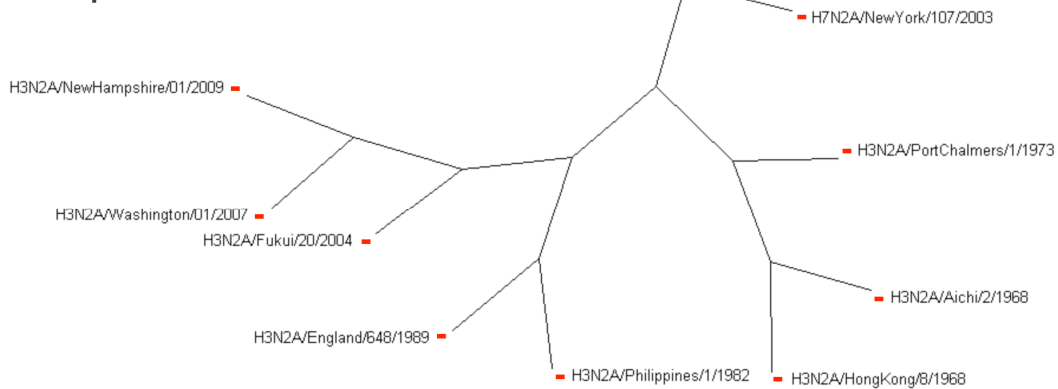

Supplement: Figure S2 — Influenza hemagglutinin unrooted phylogenetic tree of all the viral strains tested in neutralization assays with PN-SIA49. Viral isolates belonging to group 1 and group 2 are divided into two different boxes. Subtypes that can be neutralized by PN-SIA49 are indicated with a green ‘+’, while the ones that cannot be neutralized are indicate with a red ‘−’. As reported in the text, PN-SIA49 is able to neutralize all of the group 1 viruses tested in this study except for the H9N2 strain. No neutralizing activity was detected against the H3N2 viruses tested. * The recombinant HA from A/South Carolina/1/1918 (H1N1) pandemic strain was previously shown to be bound by PN-SIA49 [26], [27]. # H1N1 A/New Caledonia/20/1999 was previously shown to be neutralized by PN-SIA28 as Fab fragment [26], [27]. (PDF) [file pone.0034415.s002.pdf]

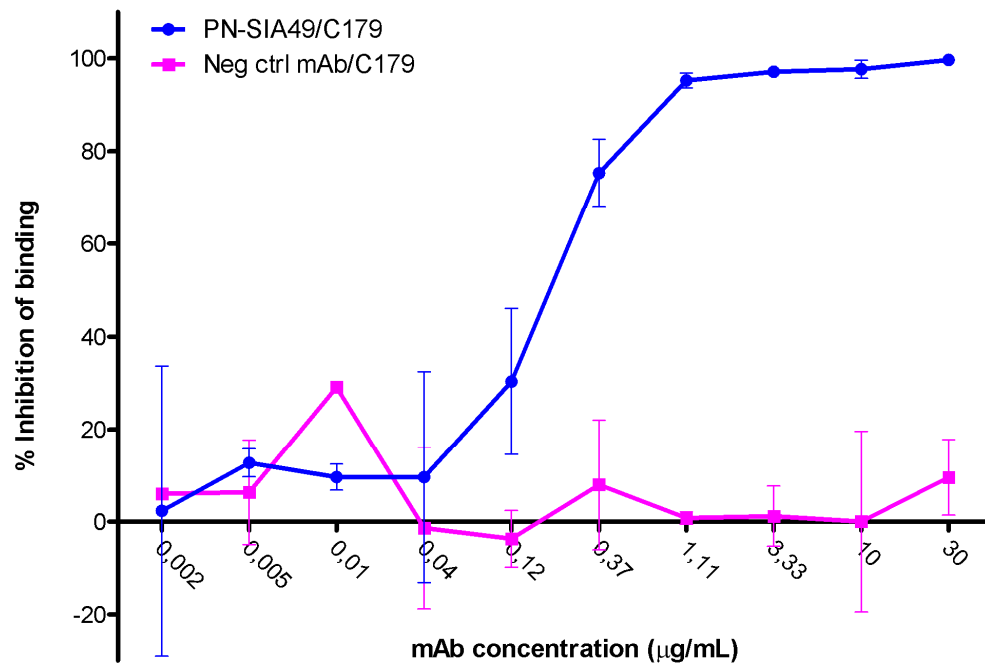

Supplement: Figure S3 — C179/PN-SIA49 competition assay. Graphic representation of cell staining and flow cytometric analysis of HEK293T cells transfected with the pcDNA 3.1D/V5-His-TOPO vector containing the HA-A/PR/8/34 were performed. Serial dilutions of PN-SIA49 were used in combination with a fixed concentration (1 µg/ml) of C179 (blue line). A monoclonal antibody directed against the HA globular head was used as competition negative control (pink line). (PDF) [file pone.0034415.s003.pdf]

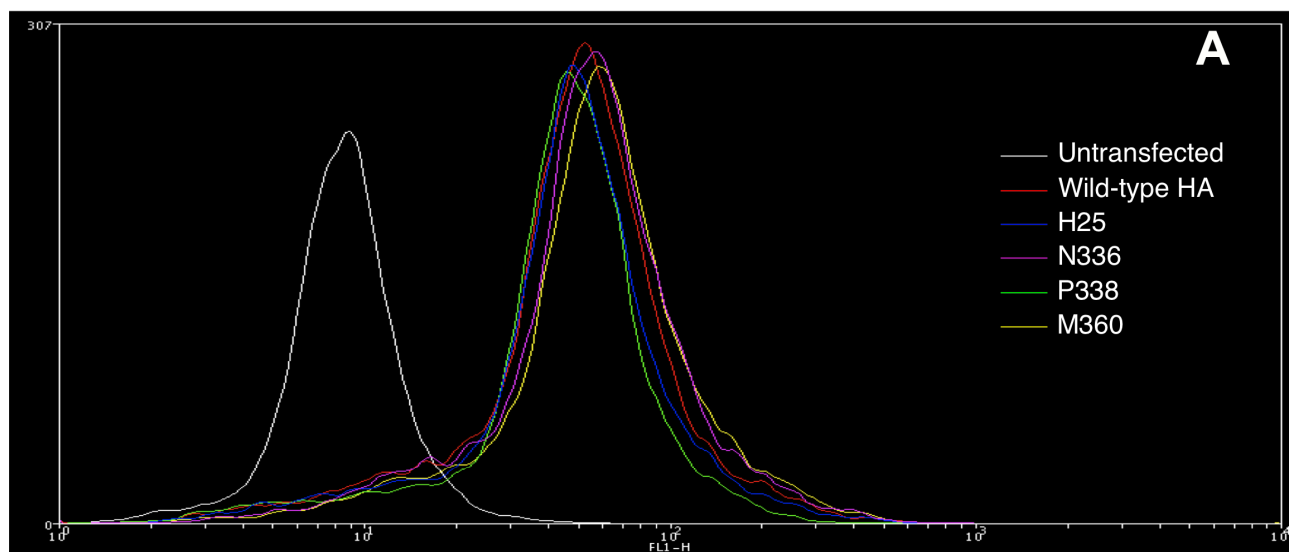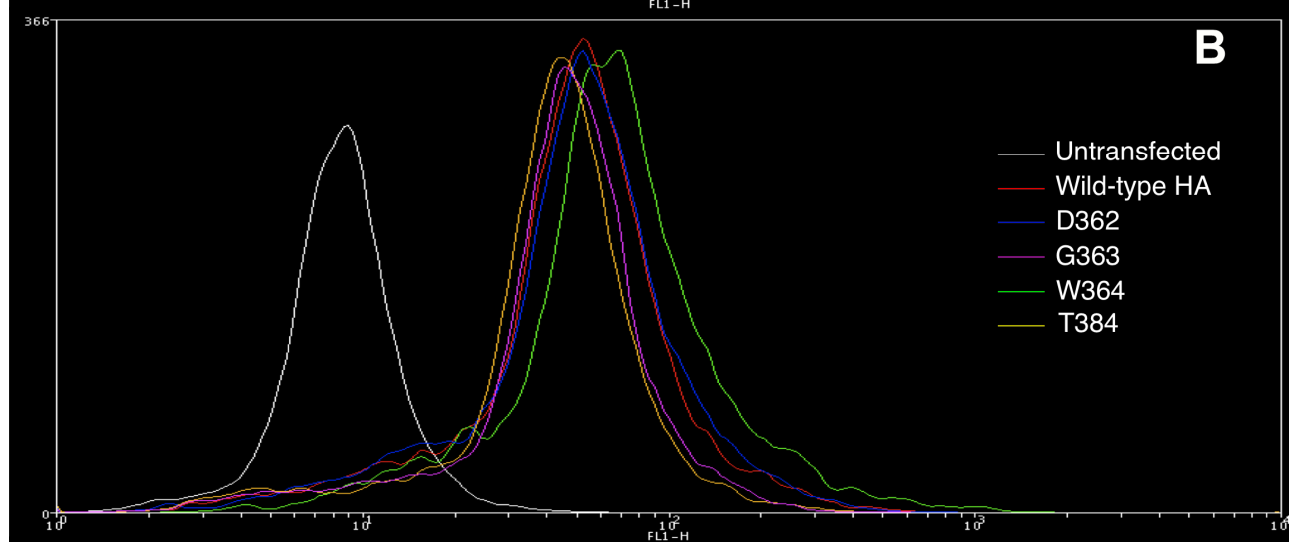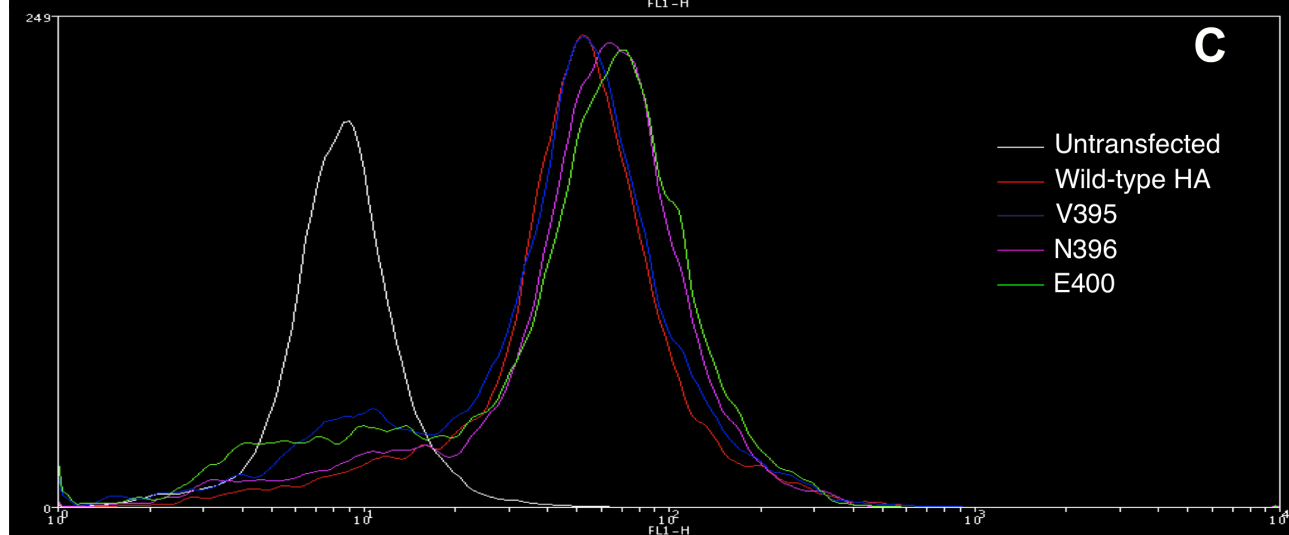

Supplement: Figure S4 — HA mutants that determine a decrease of PN-SIA49 binding to HA are expressed at the same level of wild type HA on cell surface. FACS curves showing the binding of anti-H1N1 HA antibody (directed against a linear epitope) to untransfected cells, HA wild-type and HA-mutants. White and red curves represent, for each graph, respectively the binding of anti-HA expression control to untransfected cells and wild type H1N1-HA. The different colour curves represent the different mutants. (PDF) [file pone.0034415.s004.pdf]
