# Supplementary material for: A Non-VH1-69 Heterosubtypic Neutralizing Human Monoclonal Antibody Protects Mice against H1N1 and H5N1 Viruses
Source: PLoS One. 2012 Apr 4;7(4):e34415. doi: 10.1371/journal.pone.0034415 (PMC3319592; doi:10.1371/journal.pone.0034415)
Supplement: Table S1 — Major anti-influenza human monoclonal antibodies with heterosubtypic neutralizing activity. (DOC) [file pone.0034415.s005.doc]

**Table S1.** Major anti-influenza human monoclonal antibodies with heterosubtypic neutralizing activity.

| **mAb** | **Isotype** | **VH gene** | **In vitro neutralizing activity** | | **Reference** |
| --- | --- | --- | --- | --- | --- |
|  |  |  | **Viral subtype** | **(IC_50_ μg/ml)** |  |
| **PN-SIA49** | IgG1 | 3-23 | H1 | 0.1-1.1 |  |
|  |  |  | H2 | 1.1 |  |
|  |  |  | H5 | 1.1-1.9 |  |
|  |  |  | H9 | >10 |  |
|  |  |  | H3 | >10-30 |  |
|  |  |  | H7 | >10 |  |
| **A06** | IgG1 | 1-69 | H1 | 9-250^a^ | [23] |
|  |  |  | H5 | 2-11^a^ |  |
|  |  |  | H3 | >333^a^ |  |
| **CR6261** | IgG1 | 1-69 | H1 | 8.89 | [21,25] |
|  |  |  | H2 | 14.87->50 |  |
|  |  |  | H5 | 0.55-3.7 |  |
|  |  |  | H6 | 0.12 |  |
|  |  |  | H8 | 8.89 |  |
|  |  |  | H9 | 5.24 |  |
|  |  |  | H3 | >50 |  |
|  |  |  | H4 | >50 |  |
|  |  |  | H7 | >50 |  |
| **F10** | IgG1 | 1-69 | H1 | 3.12-50^b^ | [24] |
|  |  |  | H2 | 25-50^b^ |  |
|  |  |  | H5 | 6.25-25^b^ |  |
|  |  |  | H6 | 12.5-100^b^ |  |
|  |  |  | H8 | 25 |  |
|  |  |  | H9 | 12.5-25^b^ |  |
|  |  |  | H3 | >100^b^ |  |
| **FB110** | IgG3 | 3-23 | H1 | 5-22 | [20] |
|  |  |  | H2 | 7 |  |
|  |  |  | H5 | 22-36 |  |
|  |  |  | H6 | >50 |  |
|  |  |  | H9 | >50 |  |
|  |  |  | H7 | >50 |  |
| **FC41** | IgG1 | 1-69 | H1 | 31-63 |  |
|  |  |  | H2 | 25 |  |
|  |  |  | H5 | 25-40 |  |
|  |  |  | H6 | 63 |  |
|  |  |  | H9 | 9-20 |  |
|  |  |  | H7 | >50 |  |
| **FE43** | IgG1 | 1-69 | H1 | 4-40 |  |
|  |  |  | H2 | >50 |  |
|  |  |  | H5 | 25 |  |
|  |  |  | H6 | 20 |  |
|  |  |  | H9 | 4-50 |  |
|  |  |  | H7 | >50 |  |

^a^ The neutralizing titer was defined as the minimum inhibitory concentration at which the infectivity of 100 TCID_50_ of the appropriate viruses for MDCK cells was completely neutralized.

^b^ Data are reported as neutralizing titer starting from a 100 μg/ml antibody stock solution.
